# The effect of hypercortisolism treatment on dyslipidemia in Cushing syndrome: systematic review and meta-analysis

**DOI:** 10.1210/clinem/dgaf681

**Published:** 2025-12-23

**Authors:** Gianmaria Salvio, Alessandro Ciarloni, Nairus Aboud, Nicola Ambo, Monia Bordoni, Beatrice Lucchetti, Marianna Martino, Michele Perrone, Matteo Gasparroni, Giancarlo Balercia, Giorgio Arnaldi

**Affiliations:** Endocrinology Clinic, Department of Clinical and Molecular Sciences, Polytechnic University of Marche, Ancona 60126, Italy; Endocrinology Clinic, Department of Clinical and Molecular Sciences, Polytechnic University of Marche, Ancona 60126, Italy; Endocrinology Clinic, Department of Clinical and Molecular Sciences, Polytechnic University of Marche, Ancona 60126, Italy; Endocrinology Clinic, Department of Clinical and Molecular Sciences, Polytechnic University of Marche, Ancona 60126, Italy; Endocrinology Clinic, Department of Clinical and Molecular Sciences, Polytechnic University of Marche, Ancona 60126, Italy; Endocrinology Clinic, Department of Clinical and Molecular Sciences, Polytechnic University of Marche, Ancona 60126, Italy; Endocrinology Clinic, Department of Clinical and Molecular Sciences, Polytechnic University of Marche, Ancona 60126, Italy; Endocrinology Clinic, Department of Clinical and Molecular Sciences, Polytechnic University of Marche, Ancona 60126, Italy; Endocrinology Clinic, Department of Clinical and Molecular Sciences, Polytechnic University of Marche, Ancona 60126, Italy; Endocrinology Clinic, Department of Clinical and Molecular Sciences, Polytechnic University of Marche, Ancona 60126, Italy; Section of Endocrinology, PROMISE, University of Palermo, Palermo 90127, Italy

**Keywords:** cholesterol, low-density lipoprotein, high-density lipoprotein, triglycerides, transsphenoidal neurosurgery, adrenalectomy, steroidogenesis inhibitors, pasireotide, ketoconazole, levoketoconazole, osilodrostat, mifepristone

## Abstract

**Introduction:**

Cushing syndrome (CS) is a clinical condition caused by increased plasma cortisol levels and characterized by high cardiovascular mortality. Among the metabolic effects of CS and its treatment, glycemic disturbances have been investigated in depth, while data on dyslipidemia is still lacking.

**Objectives:**

Our study aims at evaluating the effects of CS treatment on serum lipid levels.

**Materials and methods:**

A literature search was conducted using PubMed, Scopus, and EMBASE databases to investigate the effects of CS treatment on serum total cholesterol (TC), low-density lipoprotein cholesterol, high-density lipoprotein cholesterol, and triglycerides. Before-after analysis and subgroup analysis were performed.

**Results:**

Twenty-nine observational or interventional studies (51.7% of good quality) were included in the quantitative analysis. Treatment of CS led to clinically and statistically significant decrease in serum TC (mean difference [MD] −26.49; 95% CI, −29.95 to −23.04; *P* < .00001), low-density lipoprotein cholesterol (MD −18.44; 95% CI, −21.30 to −15.57; *P* < .00001), and triglycerides levels (MD −17.77; 95% CI, −22.70 to −12.84; *P* < .00001), with no significant changes in high-density lipoprotein cholesterol levels (MD −2.34; 95% CI, −6.96 to 2.28; *P* = .32). Subgroup analysis showed greater decrease in TC levels in subjects with adrenal hypercortisolism, in those treated with steroidogenesis inhibitors, and in those with treatment duration ≥12 months. In addition, CS treatment significantly decreased blood glucose levels, body mass index, waist circumference, and insulin resistance index.

**Conclusion:**

Our study demonstrates a significant improvement in serum lipid levels after treatment of CS. Because the cardiovascular complications of hypercortisolism depend on several factors, further studies are needed to determine whether this directly translates into an adequate reduction in the risk of major cardiovascular events.

Cushing syndrome (CS) is a heterogeneous condition determined by chronic excess of endogenous or exogenous glucocorticoids (GCs). CS induces a systemic and metabolic disorder characterized by visceral obesity, insulin resistance, hyperglycemia and diabetes, hypertension, dyslipidemia, and coagulation disorders leading to increased mortality, mainly due to cardiovascular disease and thromboembolism (1). Management of endogenous CS includes surgical (pituitary, ectopic, or adrenal), pharmacological, or radiotherapy approaches alone or in combination.

According to the Endocrine Society guidelines, because dyslipidemia is very common in CS—with a prevalence of 38% to 71% (1)—the lipidic profile must be evaluated at diagnosis and periodically after in all cases of endogenous hypercortisolism. Unfortunately, it is difficult to know its exact prevalence, as definitions and cutoffs for dyslipidemia vary between studies, and diagnostic criteria are also variable over time in accordance with the ongoing redefinition of the concept of cardiovascular risk. Dyslipidemia can also be influenced by other factors such as obesity and diabetes, which are frequently present in these patients (1, 2).

GCs lead to dyslipidemia with elevated levels of triglycerides (TG), total cholesterol (TC), and low-density lipoprotein cholesterol (LDL-c) by stimulating preadipocyte differentiation (2, 3). They can also stimulate cholesterol and fatty acid synthesis by the liver, potentially leading to hepatic steatosis (4). CS-related dyslipidemia is particularly threatening because it coexists with metabolic syndrome in many of these patients. If the negative effects of hypercortisolism on blood pressure, visceral obesity, and glucose metabolism are well known, patients affected by CS must be considered at even higher cardiovascular risk because of the prothrombotic effect of GCs (5-10). A prompt treatment of dyslipidemia is therefore mandatory in these patients. Several medications used for the treatment of CS have important effects on lipids. In further details, mitotane increases cholesterol and TG levels (11), whereas ketoconazole inhibits cholesterol biosynthesis and reduces apolipoprotein B and LDL-c levels by approximately 25% (12).

Although a positive impact on lipid profile is expected after hypercortisolism reversion or control, there is a lack of randomized controlled trials (RCTs) on this topic and available evidence is not always unambiguous.

The aim of this systematic review and meta-analysis is to evaluate the impact of CS treatment options on lipidic profile in endogenous hypercortisolism of different etiologies.

## Materials and methods

This study was conducted following the guidelines of The Preferred Reporting Items for Systematic reviews and Meta-Analyses (PRISMA) statement (13). The research was registered on PROSPERO (https://www.crd.york.ac.uk/prospero/) with number CRD42023437417.

### Search strategy

A systematic search was conducted through Scopus, PubMed, and EMBASE databases from June 2023 to February 2025. The following terms were combined: “Cushing”, “hypercortisolism”, “high cortisol”, “glucocorticoid excess”, “visceral fat”, “obesity”, “obese”, “adiposity”, “dyslipidemia”, “lipid”, “cholesterol”, “triglyceride”, “metabolic syndrome”, “LDL”, “HDL”, “fat”, and “lipoprotein”. Full details about research strings are provided in the Supporting materials. The search was conducted independently by 2 authors (G.S. and A.C.), and disagreements were resolved through discussion with a third author (G.A.).

### Selection criteria

The eligible studies were selected following the PICO model: Population (P: subjects with CS), Intervention (I: surgical and medical treatment of CS), Comparison (C: same patients before-after treatment), Outcome (O: the primary outcome was serum lipid levels, whereas the secondary outcomes were anthropometry and glucose metabolism). Randomized controlled trials, retrospective and prospective cohort studies, and uncontrolled studies were included. No restrictions on language were applied.

### Data extraction and quality assessment

Four authors (N.A., M.G., B.L., and M.P.) divided into pairs performed data extraction independently, and their work was verified by a fifth author (G.S.). The following data were collected: first author, year, country, study design, characteristics of patients, previous treatments, actual CS therapy, actual assumption of lipid-lowering agents, length of follow-up, age, weight, body mass index (BMI), waist circumference (WC), serum levels of TC, LDL-c, high-density lipoprotein cholesterol (HDL-c), TG, blood glucose (BG), blood insulin, calculated homeostasis model assessment (HOMA), and glycated hemoglobin (HbA1c). Sample size, mean value, and SD of continuous variables at baseline and follow-up were identified and extracted. Whenever these data were not directly available, but were presented in graphs, they were derived using PlotDigitizer software (https://plotdigitizer.com/). Whenever only standard error of the mean (SEM) was available, SD was calculated as SEM * √ (sample size). If data at baseline were present but data at follow-up were only presented as the mean change, the mean posttreatment value was calculated by summing the mean value at baseline and the mean change, and the posttreatment SD was estimated using the following formula:


$$SD_{\mathrm{post}}=\sqrt{{\mathrm{SD}}_{\mathrm{change}}^{2}-{\mathrm{SD}}_{\mathrm{pre}}^{2}+2r\cdot SD_{\mathrm{pre}}\cdot SD_{\mathrm{post}}}$$

When the value of correlation (*r*) was not known, a conservative approach was chosen by assigning a value of 0.5 (14). The quality of evidence (QoE) was assessed using the Newcastle-Ottawa Scale (NOS) for case-control and cohort studies (15), the Version 2 of the Cochrane risk-of-bias tool (RoB 2) (14) for RCTs, and the methodological index for nonrandomized studies (MINORS) tool for uncontrolled studies (16). In more detail, the NOS for case-control studies includes 8 items (Q1: Case definition; Q2: Representativeness of the cases; Q3: Selection of controls; Q4: Definition of controls; Q5: Comparability of cases and controls; Q6: Ascertainment of exposure; Q7: Same method of ascertainment; Q8: Nonresponse rate), as does the NOS for cohort studies (Q1: Representativeness of the exposed cohort; Q2: Selection of the nonexposed cohort; Q3: Ascertainment of exposure; Q4: Demonstration that outcome of interest was not present at start of study; Q5: Comparability of cohorts; Q6: Assessment of outcome; Q7: Length of follow-up; Q8: Adequacy of follow-up of cohorts), with total score ranging 0 to 9 (15). The Cochrane RoB 2 explores 5 different domains as potential source of bias, including (D1) bias due to randomization process, (D2) deviation from intended intervention, (D3) missing outcome data, (D4) measurement of outcomes, and (D5) selection of the reported results, with an “overall risk of bias” judgment (14). Finally, the MINORS tool provides 8 items (Q1: A stated aim of the study; Q2: Inclusion of consecutive patients; Q3: Prospective collection of data; Q4: Endpoint appropriate to the study aim; Q5: Unbiased evaluation of endpoints; Q6: Follow-up period appropriate to the major endpoint; Q7: Loss to follow up not exceeding 5%; Q8: prospective calculation of the study size), with each item scored 0 (not reported), 1 (reported but inadequate) or 2 (reported and adequate) and total score ranging 0 to 16. Two authors (G.S. and A.C.) independently performed the QoE, and any disagreements were resolved through discussion.

### Statistical analysis

The analysis was performed using RevMan software v. 5.4.1 (Cochrane Collaboration, Oxford, UK) and Comprehensive Meta-Analysis v. 3.7 (Biostat Inc., Englewood, USA). Mean difference (MD) or standardized mean difference and 95% CIs were calculated to compare outcome measures after treatment. *I*^2^ statistic was applied to explore heterogeneity, with *I*^2^ > 50% and *P* < .1 indicating high between-study heterogeneity. If significant heterogeneity emerged, meta-analysis was performed using a random-effects model. Otherwise, a fixed-effects model was used. If more than 1 longitudinal measurement was available, the longest follow-up from each study was selected, as recommended in the Cochrane Handbook for Systematic Reviews for Interventions (14). Publication bias was assessed by funnel plot asymmetry as well as Egger's test. Sensitivity analysis (omitting each single study to explore its effect on the overall meta-analysis) and subgroup analysis were conducted. Statistical significance was set at 0.05.

## Results

### Study selection

**Figure 1.**
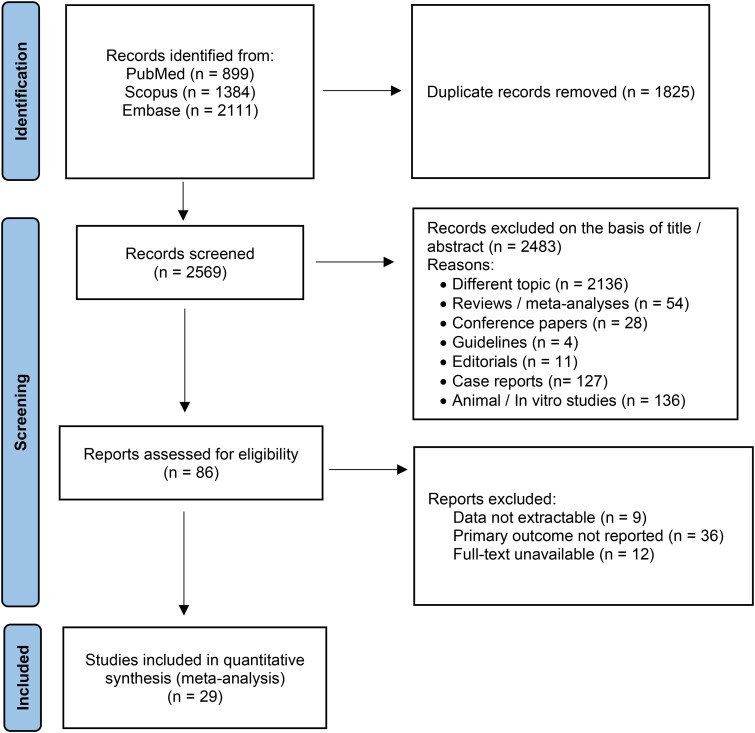
Preferred reporting items for systematic review and meta-analysis protocols (PRISMA-P) flowchart.

Using the previously mentioned search strategy, 4394 abstracts were extracted. After the removal of 1825 duplicates, 2569 articles were screened. Of these, 2483 were identified by title or abstracts as papers on other topics, review articles, editorials, case reports, animal or in vitro studies, guidelines, or congress papers. Of the remaining 86 full-text articles assessed for eligibility, 29 (5, 17-44) were included in the present meta-analysis (Fig. 1). The main characteristics of the included studies are presented in Tables 1 and  2.

**Table 1.** Characteristics of the included studies

First author	Year	Country	Study design	Sample size (treated)	Type of CS	Gender (M/F)	Treatment	Previous treatment	Controls	Follow-up time (months)	Disease control (n (%)), assessment
Akaza (17)	2010	Japan	Cohort study	21 (12)	4 CD 4 Ectopic 13 AA	4/17	7 adrenalectomy 2 TNS 1 ectopic surgery 2 ectopic metyrapone	Not stated	12 age- and gender-matched subjects	Median 8 (3-30)	12 (100), Serum morning cortisol
Albani (18)	2018	Italy (multicenter)	Prospective uncontrolled	16	CD	5/11	Pasireotide 1200-1800 mcg/daily	16 surgery 10 medication 4 irradiation	—	6-12	16 (100), UFC ≤ ULN
Colao (35)	2012	Global (multicenter)	Double-blind, randomized, dose-comparison controlled	162	CD	36/126	Pasireotide 1200 mcg/daily (82) and 1800 mcg/daily (80)	128 surgery 78 medication 7 irradiation	1200 mcg vs 1800 mcg daily	12	33/78 (42.3), UFC ≤ ULN
Debillon (19)	2015	France (multicenter)	Retrospective uncontrolled	15	PBMAH	3/12	Unilateral adrenalectomy	Not stated	—	12	15 (100), UFC ≤ ULN
Dhingra (20)	2019	India	Retrospective uncontrolled	100	73 CD 12 Adrenal 7 Ectopic 8 Unknown	33/67	Surgery	Not stated	—	3	47 (47), serum cortisol (8 Am) < 137 nmol/L and/or restoration of circadian rhythm and/or DEXA suppression < 2 mcg/dL
Erbil (21)	2006	Türkiye	Retrospective case-control	39 (28)	Not stated	2/26	Surgery	Not stated	Subclinical CS	12	Not stated
Faggiano (22)	2003	Italy	Cohort study	25	CD	8/17	25 Surgery 6 Irradiation 3 Re-intervention	—	32 sex- and age-matched subjects, 32 BMI-matched subjects	12	25 (100), UFC and ACTH ≤ ULN + normalization of serum cortisol and restoration of circadian rhythm + suppression of urinary and serum cortisol after low-dose DEXA test
Fleseriu (42)	2012	U.S. (multicenter)	Prospective uncontrolled	50	43 CD 4 Ectopic 3 Adrenal carcinoma	15/35	Mifepristone 300-1200 mg/day	42 unsuccessful pituitary surgery, 18 pituitary irradiation	—	6	Not stated
Fleseriu (23)	2016	Global (multicenter)	Cohort study (LINC 2)	19	CD	5/14	Osilodrostat 4-20 mg/day	Not stated	Follow-up cohort of LINC 1 (n = 4) and newly enrolled patients (n = 15)	5.5	15/19 (78.9), UFC ≤ ULN (controlled responders) or UFC < ULN and with ≥50% reduction from baseline (partially responders)
Fleseriu (40)	2019	Global (multicenter)	Prospective uncontrolled (SONICS)	94	80 CD 1 Ectopic 8 Adrenal 5 Unknown	17/77	Levoketoconazole 300-1200 mg/day	65 surgery 11 medication 9 irradiation 26 none	—	0,5-5 + 6	29 (31), UFC ≤ ULN
Gadelha (44)	2022	Global (multicenter)	Randomized, double-blind, placebo-controlled (LINC 4)	73 (48)	CD	5/43	Osilodrostat 4-40 mg/day	41 surgery 26 medication 6 irradiation	Placebo	3 (+ 9 open label)	37 (77), UFC ≤ ULN
Geer (24)	2012	United States	Prospective uncontrolled	14	CD	2/12	TNS	1 surgery	—	20.1	14 (100), UFC ≤ ULN
Giordano (5)	2011	Italy	Cohort study	58 (29)	14 CD 15 AA	3/26	TNS + Adrenalectomy	Not stated	Sex-, age-, and BMI-matched subjects	12+	29 (100), UFC ≤ ULN, serum cortisol below or within normal range, circadian rhythm, low-dose DEXA suppression
Guarnotta (25)	2018	Italy	Case-control study	20 surgery 10 pasireotide	CD	Surgery NA Pasireotide 2/8	Transsphenoidal surgery Pasireotide 1200-1800 mcg/day	Pasireotide patients underwent unsuccessful surgery	10 patients with persistent CD vs 20 gender- and age-matched naïve CD patients undergoing surgery	12	18/20(90), UFC ≤ ULN
Hacioglu (26)	2024	Türkiye	Cohort study	25	9 CD 13 AA 3 Adrenal hyperplasia	5/20	TNS + adrenalectomy	Not stated	Age-, gender-, and BMI-matched controls	12+	25 (100), not stated
Lacroix (39)	2020	Global (multicenter)	Prospective, randomized, double-blind controlled NCT01374906	150	CD	119/31	10 mg Pasireotide LAR (n = 74) or 30 mg Pasireotide LAR (n = 76)	Not stated	Different pasireotide doses	12	45/104 (43.3), UFC ≤ ULN
Libè (27)	2015	Italy	Prospective cohort study	14	CD	0/14	TNS	Not stated	Age- and BMI-matched healthy women	10·2 ± 0·7	14 (100), cortisol levels < 140 nmol/L
Makri (28)	2019	United States	Retrospective uncontrolled	33	CD	11/22	TNS	Not stated	—	12+	33 (100), midnight cortisol < 121 nmol/L and UFC ≤ ULN
Petersenn (36)	2017	Global (multicenter)	Randomized, double-blind, uncontrolled (NCT00434148 Extension)	162 (58)	CD	36/126	Pasireotide 300-2400 mcg/day	Not stated	—	60	11/16 (68.8), UFC ≤ ULN
Pivonello (37)	2014	Global (multicenter)	Randomized, double-blind, controlled (NCT00434148)	162	CD	36/126	1200 mcg/day Pasireotide (n = 82) or 1800 mcg/day Pasireotide (n = 80)	Not stated	Different pasireotide doses	12	29 (17.9) controlled (UFC ≤ ULN), 17 (10.5) partially controlled (UFC > ULN but with ≥ 50% reduction from baseline)
Pivonello (30)	2019	Italy (multicenter)	Prospective uncontrolled	32	CD	7/25	Pasireotide 600-1200 mcg/day	25 Surgery 9 Radiotherapy 20 Medication	—	6	19 (59.4) Full normalization (UFC < ULN) 2 (6.3) near normalization (UFC ≤ 1.1 ULN)
Pivonello (43)	2020	Global (multicenter)	Randomized, double-blind uncontrolled with a placebo-controlled withdrawal phase (LINC 3)	137	CD	31/106	Osilodrostat 4-60 mg/day	120 Surgery 102 Medication 22 Irradiation	Placebo during randomized withdrawal phase	12	104 (75.9) complete response (UFC ≤ ULN), 13 (9.5) partial response (mUFC > ULN but >50% reduction)
Pivonello (29)	2021	Global (multicenter)	Prospective, uncontrolled (SONICS sub-analysis)	94	80 CD 1 Ectopic 8 Adrenal 5 Unknown	17/77	Levoketoconazole 300-1200 mg/day	Not stated	—	6+	29/61 (48), UFC ≤ ULN
Pivonello (41)	2022	Global (multicenter)	Randomized, double-blind, placebo-controlled withdrawal study	84 (79)	70 CD 8 Adrenal 2 Ectopic 4 Unknown	20/64	Levoketoconazole 300-1200 mg/day	47/70 CD pituitary surgery	Placebo during randomized withdrawal phase	3.5-4.75 (14-19 weeks)	44 (55.7), UFC ≤ ULN
Schopohl (38)	2015	Global (multicenter)	Cohort study (NCT00434148 Extension)	162 (58)	CD	7/51	1200 mcg/day Pasireotide 1200 (n = 26) or 1800 mcg/day (n = 32)	43 Surgery 27 Medication 6 Irradiation	Different pasireotide doses	24	20 (34.5), UFC ≤ ULN
Simeoli (31)	2020	Italy	Prospective, uncontrolled	8	CD	1/7	Pasireotide 1200-1800 mcg/day	Not stated	—	12	2/7 (28.6) complete response (UFC ≤ ULN), 4/7 (57.1) partial response (UFC > ULN but >50% reduction)
Sun (32)	2021	China	Retrospective, Case-control	104 (71) → 71	CD	19/85	TNS	Previous surgery excluded	Responders vs nonresponders	12+	65 (91.5), UFC ≤ ULN
Taskinen (33)	1983	Finland	Cohort study	22 (11→ 9)	5 AA 6 CD	0/11	TNS or uni-/bilateral adrenalectomy	Not stated	Weight-matched healthy women	12	Not stated
Wonglhaw (34)	2024	Thailand	Uncontrolled, retrospective	45	AA	1/44	Unilateral adrenalectomy	Not stated	—	12	Not stated

Abbreviations: AA, adrenal adenoma; BMI, body mass index; CD, Cushing disease; CS, Cushing syndrome; DEXA, dexamethasone; IQR, interquartile range; NA, not available; PBMAH, primary bilateral macronodular adrenal hyperplasia; TNS, transsphenoidal surgery; UFC, urinary free cortisol; ULN, upper limit of normal.

**Table 2.** Characteristics of the included studies

First author	Year	Age (years) Mean ± SD Median (IQR)	BMI (kg/m^2^)	Prevalence of dyslipidemia (n, %)	Treated dyslipidemia (n, %)	Prevalence of overweight or obesity (n, %)	Prevalence of diabetes (n, %)	Prevalence of hypertension (n, %)
Akaza (17)	2010	55 ± 14	23.7 ± 4.7	8 (38.1)	4/21 (19.1)	—	12 (57.1)	19 (90.5)
Albani (18)	2018	43.8 ± 12.1	34.43 ± 12.46	8 (50.0) → 5 (31.3)	1/16 (6.3)	—	7 (43.8)	9 (56.3)
Colao (35)	2012	40	—	—	—	—	55 (34.0)	—
Debillon (19)	2015	49.6 ± 12.3	27	9 (60.0)	4/15 (26.7)	8 (53.3) overweight 5 (33.3) obese	6 (40.0)	11 (73.3)
Dhingra (20)	2019	29.7 ± 10.4	29.5 ± 5.9	65 (65.0)	—	3 (3.0) overweight 33 (33.0) obese	20 (20.0)	36 (36.0)
Erbil (21)	2006	43 ± 12	38.2 ± 3.2	11 (39.3)	5/28 (17.9)	13 (46.4) obese	14 (50.0)	17 (60.7)
Faggiano (22)	2003	34.2 ± 1.9	29.2 ± 1.80	13 (52.0)	—	12 (48.0) overweight 8 (32.0) obese	5 (20.0)	18 (72.0)
Fleseriu (42)	2012	45.4 ± 11.9	35.7 ± 9.90	—	—	—	29 (58.0)	40 (80.0)
Fleseriu (23)	2016	36.8 ± 8.4	30.7 ± 7.0	6 (31.6)	—	—	8 (42.1)	13 (68.4)
Fleseriu (40)	2019	43.7 ± 13.4	30.8 ± 8.2	34 (36.2)	—	—	36 (38.3)	67 (71.3)
Gadelha (44)	2022	41.0 (21.0-67.0)	—	—	—	—	—	—
Geer (24)	2012	38.2 ± 15.1	32.1 ± 5.6	—	2 (14.3)	4 (28.6) overweight 6 (42.9) obese	2 (14.3)	13 (92.9)
Giordano (5)	2011	39.6 ± 3.2	28.9 ± 1.0	17 (58.6)	2 (6.9)	11 (37.9) overweight 11 (37.9) obese	7 (24.1)	21 (72.4)
Guarnotta (25)	2018	PAS 43.2 ± 11.8	S 29.3 ± 3.9 P 38.6 ± 9.8	—	—	—	PAS 8/10 (80.0)	—
Hacioglu (26)	2024	40.6 ± 14.0	33.34 ± 5.73	4 (16.0)	0 (0.0)	—	10 (40.0)	13 (52.0)
Lacroix (39)	2020	38.5	29.0 ± 6.7	57 (37.9)	32 (21.3)	46 (30.7) overweight 56 (37.3) obese	22 (14.7)	110 (73.3)
Libè (27)	2015	39.5 ± 14.6	25.8 ± 5.2	—	—	—	—	—
Makri (28)	2019	13.8 ± 4.0	—	—	—	—	—	—
Petersenn (36)	2017	40.2	—	20 (12.3)	—	—	36 (22.2)	18 (11.1)
Pivonello (37)	2014	40.2	30.3 ± 7.0	29 (17.9)	—	—	55 (33.9)	126 (77.7)
Pivonello (30)	2019	47	34.1 ± 10.4	16 (61.5)	5 (15.4)	8 (30.8) Overweight 15 (57.7) Obese	9 (34.6)	16 (61.5)
Pivonello (43)	2020	40.0 (31.0-49.9)	30.3 ± 7.8	—	21 (15.3)	—	27 (19.7)	25 (18.2)
Pivonello (29)	2021	DM 48.3 ± 12.3 nDM 40.8 ± 13.5	DM 33.5 ± 6.7 nDM 27.8 ± 6.4	34 (36.2)	—	—	36 (38.3)	67 (71.3)
Pivonello (41)	2022	44.7 ± 12.7	31.0 ± 6.8	—	—	—	35 (41.7)	68 (80.9)
Schopohl (38)	2015	40.9	—	—	—	—	—	—
Simeoli (31)	2020	38.9 ± 17.6	27.7 ± 6.3	6 (75.0)	1 (12.5)	3 (37.5) Overweight 2 (25.0) Obese	1 (12.5)	6 (75.0)
Sun (32)	2021	33.0 ± 11.7	26.6	23 (32.4)	—	—	—	—
Taskinen (33)	1983	34	—	—	—	—	—	—
Wonglhaw (34)	2024	44.2 ± 14.7	25.9 ± 5.4	27 (60.0)	—	26 (57.8) Obese	14 (31.1)	33 (73.3)

Abbreviations: BMI, body mass index; CD, Cushing disease; DM, diabetes mellitus; IQR, interquartile range; nDM, no diabetes mellitus; PAS, pasireotide.

### Quality assessment

Among the 11 controlled studies, 3 (21, 25, 32) were case-control studies of good quality, whereas 8 were cohort studies, of which 7 (5, 17, 22, 23, 26, 27, 38) were of good quality and 1 (33) was of fair quality. Fourteen studies did not have a control group, and of these 11 (18, 24, 28-31, 34, 36, 40-42) were of moderate quality, 1 (43) of good quality, and 2 (19, 20) of low quality. The remaining 4 studies (35, 37, 39, 44) were all RCTs of good quality. The results of the quality assessment are shown in detail in Table 3.

**Table 3.** Quality assessment for the included studies

Study	Assessment tool	Q1	Q2	Q3	Q4	Q5	Q6	Q7	Q8	Score (of 9)	Quality*^a^*
Erbil et al (2006) (21)	NOS for case-control studies	★	★	★	—	★	★	★	—	6	**(+)**
Guarnotta et al (2018) (25)	NOS for case-control studies	★	—	—	★	★★	★	★	★	7	**(+)**
Sun et al (2021) (32)	NOS for case-control studies	★	—	★	★	★★	★	★	★	8	**(+)**

		Q1	Q2	Q3	Q4	Q5	Q6	Q7	Q8	Score (of 9)	Quality*^a^*
Akaza et al (2010) (17)	NOS for cohort studies	★	★	★	★	★	★	★	—	7	**(+)**
Faggiano et al (2003) (22)	NOS for cohort studies	—	—	★	★	★★	★	★	★	7	**(+)**
Fleseriu et al (2016) (23)	NOS for cohort studies	★	—	★	★	★	★	★	★	7	**(+)**
Giordano et al (2011) (5)	NOS for cohort studies	—	—	★	★	★★	★	★	★	7	**(+)**
Hacioglu et al (2023) (26)	NOS for cohort studies	—	—	★	★	★★	★	★	★	7	**(+)**
Libè et al (2015) (27)	NOS for cohort studies	—	—	★	★	★★	★	★	★	7	**(+)**
Schopohl et al (2015) (38)	NOS for cohort studies	★	★	★	★	★★	★	★	★	8	**(+)**
Taskinen et al (1983) (33)	NOS for cohort studies	—	—	★	—	★	★	★	—	4	**(!)**

		Q1	Q2	Q3	Q4	Q5	Q6	Q7	Q8	Score (of 16)	Quality*^b^*
Albani et al (2018) (18)	MINORS	2	0	2	2	0	2	2	0	10	**(!)**
Debillon et al (2015) (19)	MINORS	2	0	0	2	0	2	2	0	8	**(−)**
Dhingra et al (2019) (20)	MINORS	1	0	0	1	0	1	2	0	5	**(−)**
Fleseriu et al (2012) (42)	MINORS	2	2	2	2	2	2	1	0	13	**(!)**
Fleseriu et al (2019) (40)	MINORS	2	1	2	2	2	2	1	2	14	**(!)**
Geer et al (2012) (24)	MINORS	2	2	2	2	2	2	2	0	14	**(!)**
Makri et al (2019) (28)	MINORS	2	1	1	2	1	1	2	0	10	**(!)**
Petersenn et al (2017) (36)	MINORS	2	2	2	2	2	2	1	0	13	**(!)**
Pivonello et al (2019) (30)	MINORS	2	1	2	2	1	2	2	0	12	**(!)**
Pivonello et al (2020) (43)	MINORS	2	2	2	2	2	2	1	2	15	**(+)**
Pivonello et al (2021) (29)	MINORS	2	2	2	2	2	2	1	1	14	**(!)**
Pivonello et al (2022) (41)	MINORS	2	2	2	2	2	2	1	2	15	**(!)**
Simeoli et al (2020) (31)	MINORS	2	2	2	2	2	2	1	1	14	**(!)**
Wonglhaw (2024) (34)	MINORS	1	1	1	1	1	2	2	0	9	**(!)**

		D1	D2	D3	D4	D5	Overall	
Colao et al (2012) (35)	Cochrane RoB 2	Some concerns	Low risk	Low risk	Low risk	Low risk	Low risk	**(+)**
Gadelha et al (2022) (44)	Cochrane RoB 2	Low risk	Low risk	Low risk	Low risk	Low risk	Low risk	**(+)**
Lacroix et al (2020) (39)	Cochrane RoB 2	Low risk	Low risk	Low risk	Low risk	Low risk	Low risk	**(+)**
Pivonello et al (2014) (37)	Cochrane RoB 2	Low risk	Low risk	Low risk	Low risk	Low risk	Low risk	**(+)**

Abbreviations: MINORS, Methodological Index for Non-Randomized Studies; NOS, Newcastle-Ottawa Scale; RoB, risk of bias.

^*a*^Quality: **(−)** Poor quality: 0-2 **(!)** Fair quality: 3-5 **(+)** High quality: 6-9.

^*b*^Quality: **(−)** Poor quality: 8 or less **(!)** Moderate quality: 9-14 **(+)** High quality: 15-16.

### Primary outcomes

**Figure 2.**
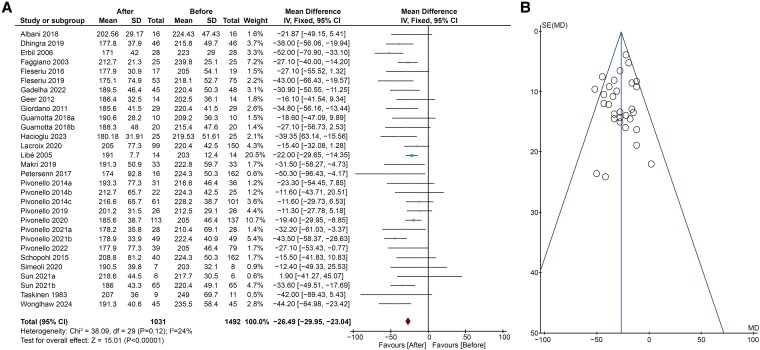
(A) Forest plot of serum TC levels of patients before and after treatment of CS; (B) Funnel plot of the included studies. Abbreviations: CS, Cushing syndrome; TC, total cholesterol.

The effects of CS treatment on TC levels were evaluated in 26 studies (5, 18, 20-29, 29-34, 36-41, 43, 44) (Fig. 2A), showing a significant decrease after therapy (MD −26.49; 95% CI, −29.95 to −23.04; *P* < .00001). Heterogeneity was low (*I*^2^ = 24%, *P* = .12) and no publication bias were found (Egger's test *P* = .352; Fig. 2B). Sensitivity analysis identified the study by Erbil et al (21) as the major source of heterogeneity, but after its removal no significant changes emerged (MD −25.61; 95% CI, −29.13 to −22.09; *P* < .00001, *I*^2^ = 9%).

**Figure 3.**
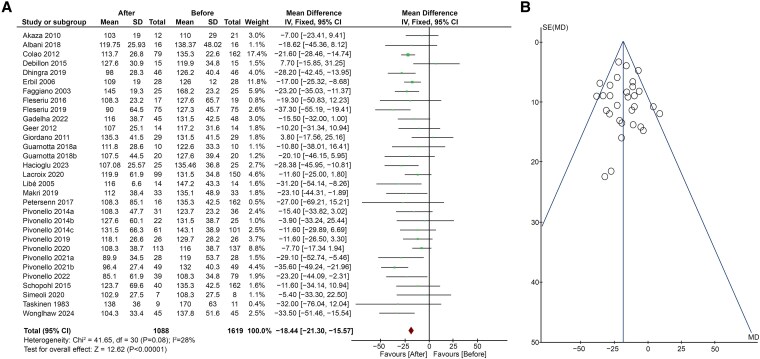
(A) Forest plot of serum LDL-c levels of patients before and after treatment of CS; (B) Funnel plot of the included studies. Abbreviations: CS, Cushing syndrome; LDL-c, low-density lipoprotein cholesterol.

The effects of CS treatment on LDL-c levels were evaluated in 27 studies (5, 17, 18, 20-41, 43, 44) (Fig. 3A). A significant decrease in LDL-c levels emerged after treatment (MD −18.44; 95% CI, −21.30 to −15.57; *P* < .00001), with low heterogeneity (*I*^2^ = 28%, *P* = .08). Egger's test (*P* = .770) and Funnel plot (Fig. 3B) showed no publication bias. Regarding sensitivity analysis, after the removal of the study by Pivonello et al (29), heterogeneity decreased slightly, but the results were unchanged (MD −17.47; 95% CI, −20.42 to −14.51; *P* < .00001; *I*^2^ = 19%).

Twenty-two studies (5, 17, 18, 20-34, 40, 42-44) reported data on HDL-c levels before-after treatment (Figure S1A) (45), showing that CS treatment had negligible effects on HDL-c levels (MD −2.34; 95% CI, −6.96 to 2.28; *P* = .32), with high heterogeneity (*I*^2^ = 94%, *P* < .00001). Moreover, Egger's test (*P* = .005) and Funnel plot (Figure S1B) (45) suggested significant risk of publication bias. A slight decrease in heterogeneity emerged after the removal of the study by Libé et al (27), but results were unchanged (MD −3.32; 95% CI, −6.72 to 0.08; *P* = .006; *I*^2^ 83%).

**Figure 4.**
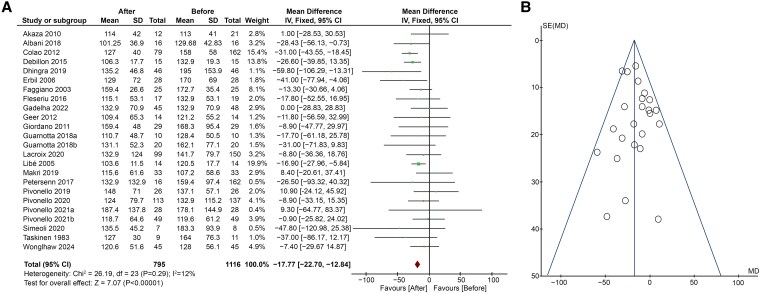
(A) Forest plot of serum TG levels of patients before and after treatment of CS; (B) Funnel plot of the included studies. Abbreviations: CS, Cushing syndrome; TG, triglycerides.

Regarding TG, 22 studies (5, 17-25, 27-31, 33-36, 39, 43, 44) showed that the treatment of CS led to a significant decrease in TG levels (MD −17.77; 95% CI, −22.70 to −12.84; *P* < .00001), with low heterogeneity among studies (*I*^2^ = 12%, *P* = .29) (Fig. 4A). No risks for publication bias were shown by Funnel plot (Fig. 4B) and Egger's test (*P* = .971). Excluding the study by Dhingra et al (20), heterogeneity decreased further (*I*^2^ = 0%), but the effects were unchanged (MD −15.36; 95% CI, −20.72 to −10.00; *P* < .00001).

### Subgroup analysis

#### Pituitary vs adrenal disease

**Figure 5.**
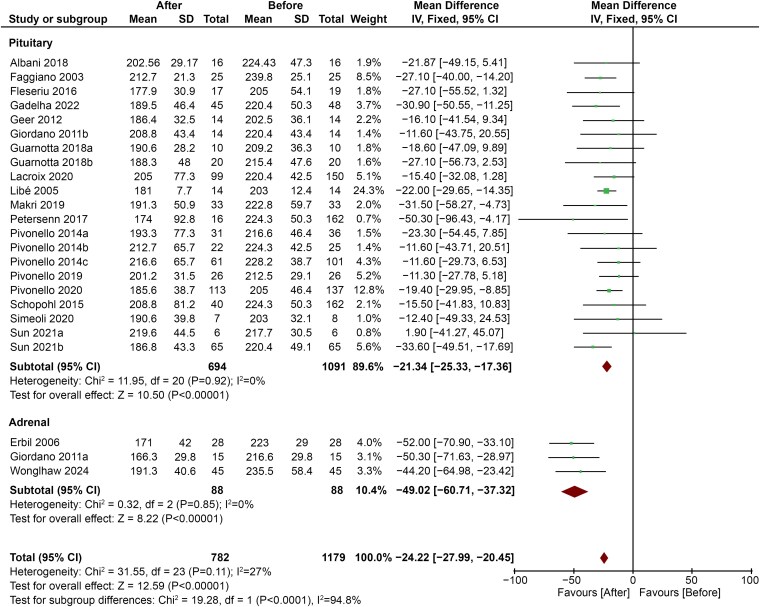
Comparison between decrease in TC levels in patients with adrenal or pituitary disease after treatment of CS. Abbreviations: CS, Cushing syndrome; TC, total cholesterol.

Subgroup analysis showed that subjects affected by adrenal disease showed a more pronounced decrease in TC levels (MD −49.02; 95% CI, −60.72 to −37.32; *P* < .00001) compared with subjects with pituitary disease (MD −21.34; 95% CI, −25.33 to −17.36; *P* < .00001) (Fig. 5).

No significant differences emerged when subgroup analysis was conducted on HDL-c levels. Both LDL-c and TG levels showed a tendency toward greater improvement in subjects with adrenal disease, but subgroup differences were not statistically significant (Figs. S2-S4) (45).

#### Surgery vs pharmacological treatment

Subgroup analysis showed no significant differences between surgery and pharmacotherapy on serum lipids in patients with CS (Figs. S5-S80) (45).

#### Pituitary-targeting drugs vs steroidogenesis inhibitors

**Figure 6.**
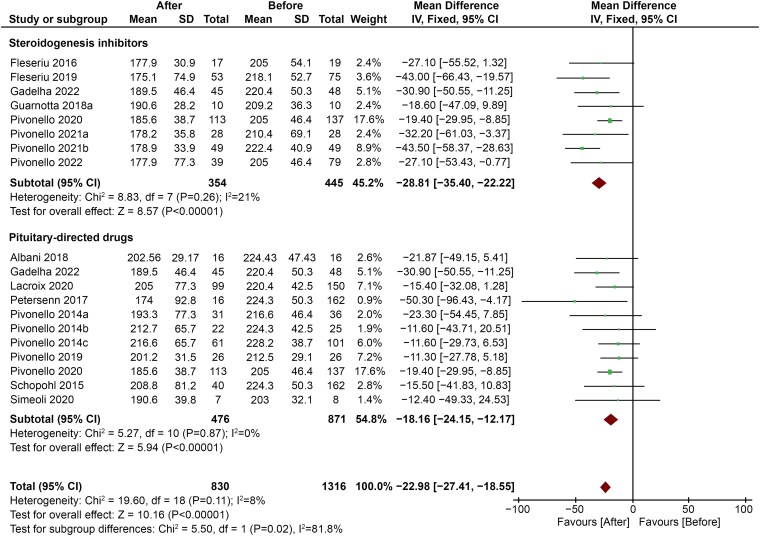
Comparison between changes in TC levels in patients treated with pituitary-targeting drugs vs steroidogenesis inhibitors. Abbreviation: TC, total cholesterol.

Subgroup analysis showed that subjects treated with steroidogenesis inhibitors showed a more pronounced decrease in TC levels (MD −28.81; 95% CI, −35.40 to −22.22; *P* < .00001) compared with subjects treated with pituitary-targeting drugs (MD −18.16; 95% CI, −24.15 to −12.17; *P* < .00001) (Fig. 6).

**Figure 7.**
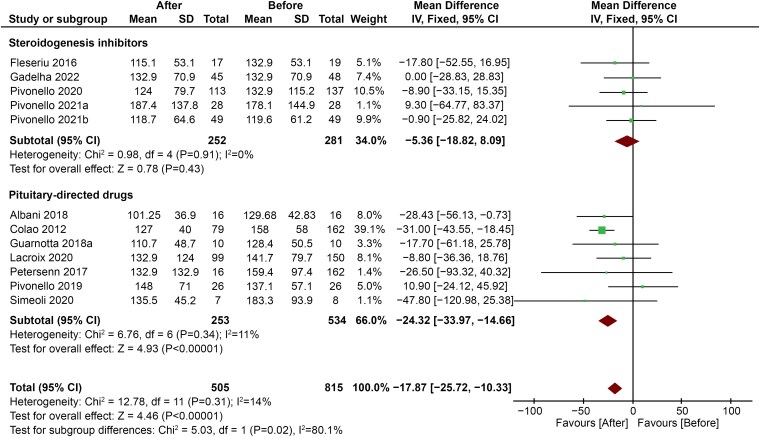
Comparison between changes in TG levels in patients treated with pituitary-targeting drugs vs steroidogenesis inhibitors. Abbreviation: TG, triglycerides.

On the opposite hand, a more pronounced decrease in TG levels emerged in subjects treated with pituitary-targeting drugs (MD −24.32; 95% CI, −33.97 to −14.66; *P* < .00001) compared with subjects treated with steroidogenesis inhibitors (MD −5.36; 95% CI, −18.82 to 8.09; *P* = .43) (Fig. 7).

HDL-c levels showed a significant decrease in subjects treated with steroidogenesis inhibitors (MD −9.47; 95% CI, −12.29 to −6.65; *P* < .00001) compared with subjects treated with pituitary-targeting drugs (MD 2.82; 95% CI, −5.41 to 11.05; *P* = .50). A negative effect was also noted for the GC antagonist mifepristone in the single study by Fleseriu et al (42) (Fig. S9) (45). On the other hand, subgroup analysis showed no significant results when comparing different types of drugs on serum levels of LDL-c (Fig. S10) (45).

#### Comparison between different durations of treatment

**Figure 8.**
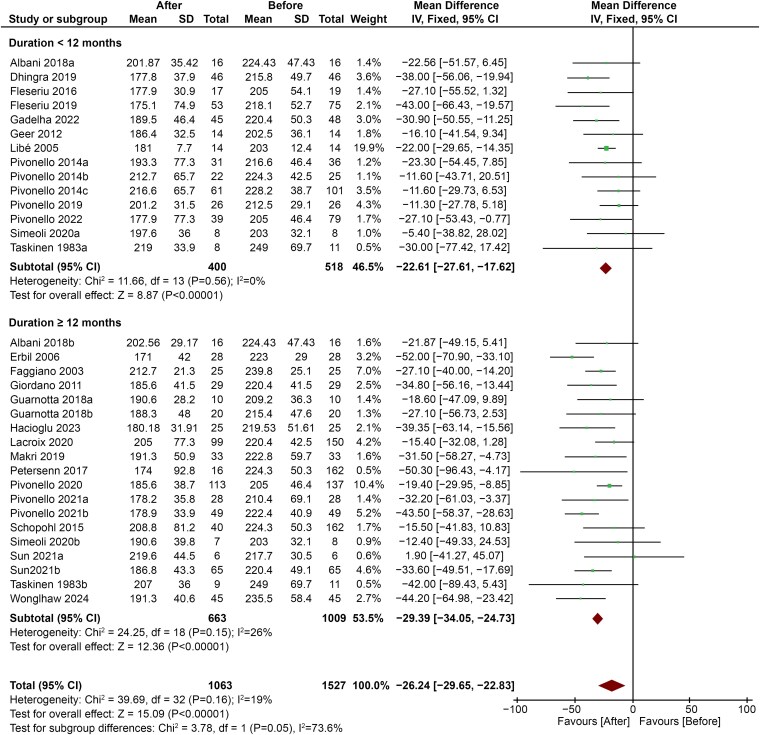
Comparison between decrease in TC levels in patients treated for ≥12 months vs patients treated for ≤12 months. Abbreviation: TC, total cholesterol.

Subgroup analysis showed that treatment duration ≥12 months led to a more pronounced decrease in TC levels (MD −29.39; 95% CI, −34.05 to −24.73; *P* < .00001) compared with shorter duration (MD −22.61; 95% CI, −27.61 to −17.62; < .00001) (Fig. 8).

Conversely, no significant differences emerged in LDL-c, HDL-c, and TG levels for different treatment durations (Figs. S11-S13) (45).

### Secondary outcomes

As secondary outcomes, the effects of CS treatment on BG (Figure S14A) (45), HbA1c (Figure S14B) (45), BMI (Figure S14C) (45), WC (Figure S14D) (45), and HOMA index (Figure S14E) (45) were evaluated. All of these parameters showed a significant improvement, except for HbA1c. In greater detail, a mean decrease of 10.66 mg/dL in BG (*P* < .00001), 2.63 kg/m^2^ in BMI (*P* < .00001), 5.68 cm in WC (*P* = .0001), and 1.62 in HOMA index (*P* = .0003) was observed.

When studies on pasireotide (18, 30, 31, 35-39) were excluded, no significant differences emerged in BG, BMI, and WC changes after treatment, but changes in HbA1c levels before-after treatment became statistically significant (MD −0.30; 95% CI, −0.41 to −0.19; *P* < .00001) (Fig. S15) (45).

## Discussion

Guidelines on medical treatment of patients with CS recommend monitoring and adjunctive treatment for cortisol-dependent comorbidities, including dyslipidemia, since glucose and lipid metabolism may improve after effective treatment of CS, but the prevalence of diabetes and dyslipidemia after remission remains higher than in BMI-matched controls (1). These data are reflected by the recent retrospective matched-cohort study by Akirov et al, who showed that mortality risk is lowered by disease remission, but remains higher in patients with CS as compared to the general population (46). Quite surprisingly, although cardiovascular disease is a leading cause of death in patients with CS (47), dyslipidemia is a relatively underinvestigated aspect.

Subjects affected by CS typically show hypercholesterolemia with high TG and LDL-c levels and low HDL-c levels (3). Indeed, hypercortisolism directly affects serum lipid levels promoting both very low-density lipoprotein secretion from the liver and lipolysis with release of free fatty acids from the adipocytes. Moreover, visceral fat increase, diabetes mellitus, and insulin resistance induced by GCs may further worsen the atherogenic lipid profile (48). The results of our meta-analysis clearly show that treatment of CS leads to dramatic improvement in lipid metabolism by lowering serum levels of TC, LDL-c, and TG, regardless of the chosen option. Conversely, HDL-c levels seem less likely to improve after therapy. Of note, even when associated with ameliorations in lipid profile, clinical and biochemical improvement of hypercortisolism may not result in complete resolution of all cardiovascular abnormalities, and it has been suggested that the cardiovascular risk of those with CS remains high even after remission (22).

In our study, patients with adrenal hypercortisolism showed a greater decrease in TC levels after treatment, when compared with subjects with Cushing disease (CD). In a recent study by Li et al (49), the metabolic profiles of patients with ACTH-independent and ACTH-dependent CS were similar, with overlapping levels of TC, LDL-c, HDL-c, and TG. Notably, their sample size was quite small (60 adrenal CS and 50 pituitary CS), patients with CS with ectopic ACTH secretion were excluded, and no data were available on 24-hour urinary free cortisol levels. Unfortunately, larger studies comparing the metabolic profiles of subjects with adrenal vs pituitary CS only focus on the prevalence of overweight, hypertension, and diabetes mellitus (50, 51), while no information is given on lipid profile. In a study comparing 14 patients with CD and 15 patients with adrenal adenomas (AAs), Giordano et al found that subjects with active hypercortisolism had similar levels of TC and LDL-c, which were both higher than in controls. Notably, HDL-c levels did not differ between subjects with CS and healthy controls, and a significant correlation was found between LDL-c and urinary free cortisol both in subjects with CD (*r* = 0.63, *P* = .01) and in those with AAs (*r* = 0.54, *P* = .04). Interestingly, 1 year after remission, TC and LDL-c remained higher in patients with CD compared with subjects with AAs, although 2 patients with CD were taking statins (5). Similarly, Mancini et al found higher, even if not significantly, levels of serum LDL-c in 27 patients with CD compared with 19 patients with adrenal CS (216.5 ± 46.4 vs 158.5 ± 28.2 mg/dL, *P* = .16) (52). Taken together, these data suggest that, despite similarities in the lipid profile between subjects with active adrenal CS and CD, after successful surgery the improvement of serum lipids could be greater in patients with adrenal disease, while the need for further treatment with lipid-lowering agents to effectively decrease cardiovascular risk is more likely in patients with CD. This finding takes on particular relevance when considering that individuals with CD are generally younger than those with ACTH-independent hypercortisolism (42.7 ± 13.5 vs 46.9 ± 13.6 years old, *P* < .05) (51), thus being exposed to the deleterious effects of hypercholesterolemia for longer.

Interestingly, the favorable effect on serum lipids seems to be independent of the approach chosen to treat hypercortisolism (surgical vs pharmacological), but different drug classes seem to exert diverse effects on the lipid profile composition. Indeed, pituitary-directed drugs were found to be less effective than steroidogenesis inhibitors in improving serum lipids. Since pasireotide had been used in all the studies included in the group of pituitary-directed drugs (18, 30, 31), it can be assumed that this effect depends, at least in part, on the known adverse metabolic effects of this compound. Indeed, pasireotide is a multireceptor-targeted somatostatin receptor ligand that collaterally impairs pancreatic insulin and intestinal glucagon-like peptide-1 secretion, with subsequent hyperglycemia and risk of overt diabetes mellitus (53). Our analysis of secondary outcomes confirmed that the improvement in glucose metabolism achieved by CS treatment is dampened by this effect. Since hyperglycemia and diabetes mellitus induce profound alterations on lipid metabolism, including increased production of very low-density lipoprotein and LDL-c, decreased HDL-c, and increased LDL oxidation (54), the expected improvement in lipid profile may also be dampened in patients treated with pasireotide. On the other hand, the use of steroidogenesis inhibitors, but not of pituitary-targeting drugs, leads to a significant decrease in HDL-c levels, with subsequent potentially unfavorable cardiovascular effects (55). This could be related to the raise in ACTH levels due to adrenal blockade (2), which can stimulate the scavenger receptor class B type 1 (SR-B1). SR-B1 is a selective HDL receptor expressed by the adrenal glands of both rodents and humans (56). Studies on individuals with low plasma HDL-c confirmed that HDL-c could play a role as cholesterol donor for adrenal steroidogenesis (57). The single study by Fleseriu et al (42) showed that the effects of GC antagonists could be even worse. The effects of mifepristone on serum HDL-c were confirmed in a double-blind, randomized, placebo-controlled trial on 30 healthy postmenopausal female volunteers, with a raise in ACTH that did not correlate with serum lipids (58). Since ACTH rises under treatment with GC antagonists (59) as well, it is possible that the lowering of HDL-c levels again depends on the shift of cholesterol in the steroidogenesis biosynthetic pathway.

A relationship between CS, insulin resistance, and alterations in carbohydrate and lipid metabolism is well known (60), and CS is accompanied by a peculiar form of sarcopenia in which insulin resistance and muscle strength are altered more than would be expected from the reduction in muscle mass alone (61). Furthermore, sarcopenia is only partially reversible after remission of hypercortisolism (62). Despite this, direct evidence on the effect of hypercortisolism correction on insulin resistance indices is extremely scarce. In fact, in our research, only 4 studies (24, 27, 41, 42) reporting the HOMA index were identified and analyzed, showing mixed results, with an overall favorable effect on insulin resistance after treatment. Similar results were reported by Libè et al (27) using the quantitative insulin sensitivity check index (QUICKI). In contrast, the study by Guarnotta et al (25) showed a nonsignificant effect on insulin resistance as assessed by the Matsuda index. Further studies are therefore needed on the relationship between correction of hypercortisolism and improvement in insulin sensitivity.

Finally, our results suggest that serum lipids may have a time-dependent decrease, since durations of treatment longer than 12 months led to greater improvement in TC levels. Although this may seem reassuring and induce a wait-and-see attitude, a study with an average follow-up duration of 12 years showed that mortality from CS is highest during the first year after diagnosis (hazard ratio 5.2; 95% CI, 2.7-9.7 vs general population) and that the likelihood of stroke and venous thromboembolism is much higher during this timeframe. Thereafter, mortality remains higher than in the general population (HR 2.3; 95% CI, 1.8 −2.9) and the risk of myocardial infarction remains high even in long-term follow-up. In addition, the risk of infarction is higher in subjects younger than 44 years than in older subjects (63).

Regarding QoE, 15 of 29 studies (51.7%) were of good quality, only 2 (6.9%) were of low quality, and the remaining were of moderate quality. However, most of the available data derives from nonrandomized or uncontrolled studies, and only 4 studies were RCTs. In this regard, it should be noted that they were all funded by pharmaceutical organizations, with a conflict of interest that could be considered a potential source of bias in the opinion of other authors (64). Unfortunately, this represents a potential limitation of our study. Similarly, the effect of gender and any associated lipid-lowering therapies are confounding factors in our analysis that deserve further investigation.

In conclusion, our systematic review and meta-analysis shows that treatment of CS globally leads to effective improvement in serum lipid levels. However, this does not automatically translate into a reduction in long-term cardiovascular risk, which instead remains increased relative to the general population even after complete remission of CS (65). Since CS is an insidious-onset disease, diagnosed with an average delay of about 3 years (mean 34 months, 30 months for adrenal CS, 38 months for CD) (66), cardiovascular comorbidities often occur before the appearance of clinically overt hypercortisolism (63). Surprisingly, CS is not taken into account as a risk factor for cardiovascular disease in the most common guidelines on management of dyslipidemias (55), even if a recent meta-analysis including >15 million subjects clearly demonstrated that patients taking GCs had a higher risk of major adverse cardiovascular events (relative risk [RR] = 1.27; 95% CI, 1.15-1.40), coronary heart disease (RR = 1.25; 95% CI, 1.11-1.41), and heart failure (RR = 1.92; 95% CI, 1.51-2.45) (67). Treatment of dyslipidemia in patients with CS should therefore be timely and aggressive. Although specific cholesterol targets are not available, the authors’ opinion is that individuals with CS should be considered to be at high to very high cardiovascular risk regardless of disease status. The type of treatment chosen and disease activity could be used to modulate the intensity of cholesterol-lowering therapies.

## Data Availability

Original data was generated for this study from data in the published literature. These data are included in this article and in the data repository listed in the References

## Abbreviations

AA: adrenal adenoma
BG: blood glucose
BMI: body mass index
CD: Cushing disease
CS: Cushing syndrome
GC: glucocorticoid
HbA1c: glycated hemoglobin
HDL-c: high-density lipoprotein cholesterol
HOMA: homeostasis model assessment
LDL-c: low-density lipoprotein cholesterol
MD: mean difference
NOS: Newcastle-Ottawa Scale
QoE: quality of evidence
RCT: randomized controlled trial
TC: total cholesterol
TG: triglycerides
WC: waist circumference
